# Extracorporeal membrane oxygenator as a bridge to successful surgical repair of bronchopleural fistula following bilateral sequential lung transplantation: a case report and review of literature

**DOI:** 10.1186/1749-8090-2-28

**Published:** 2007-06-05

**Authors:** Nouman U Khan, Mohamed Al-Aloul, Noman Khasati, Ali Machaal, Colm T Leonard, Nizar Yonan

**Affiliations:** 1 Department of Cardiothoracic Transplant, Wythenshawe Hospital, Manchester, M23 9LT, UK

## Abstract

**Background:**

Lung transplantation (LTx) is widely accepted as a therapeutic option for end-stage respiratory failure in cystic fibrosis. However, airway complications remain a major cause of morbidity and mortality in these patients, serious airway complications like bronchopleural fistula (BPF) are rare, and their management is very difficult.

**Case presentation:**

A 47-year-old man with end-stage respiratory failure due to cystic fibrosis underwent bilateral sequential lung transplantation. Severe post-operative bleeding occurred due to dense intrapleural adhesions of the native lungs. He was re-explored and packed leading to satisfactory haemostasis. He developed a bronchopleural fistula on the 14^th ^post-operative day. The fistula was successfully repaired using pericardial and intercostal vascular flaps with veno-venous extracorporeal membrane oxygenator (VV-ECMO) support. Subsequently his recovery was uneventful.

**Conclusion:**

The combination of pedicled intercostal and pericardial flaps provide adequate vascular tissue for sealing a large BPF following LTx. Veno-venous ECMO allows a feasible bridge to recovery.

## Background

Airway complications remain a major cause of morbidity and mortality after lung transplantation [1]. While the overall incidence of such complications can be as high as 15%, bronchopleural fistulas (BPF) are fortunately very uncommon. This is, however, a disastrous event after lung transplants. Most cases occur early after transplantation, are extremely difficult to treat and are associated with a high mortality. We report successful surgical repair of bronchopleural fistula in the donor bronchus employing a Levitronix Centrimag^® ^[Waltham, Massachusetts]-based veno-venous extracorporeal membrane oxygenator support with 6 days bridge to recovery.

## Case presentation

A 47-year-old man with end-stage respiratory failure due to cystic fibrosis underwent bilateral sequential lung transplantation. The donor was a 48-year-old male who died of sub-arachnoid haemorrhage and was ventilated for 24 hours. The pulmonary evaluation was normal, with a pO2 of 59.7 kPa on FiO2 of 1, normal chest X-ray and bronchoscopic appearance. The retrieval was performed with the standard technique, using both ante-grade and retro-grade flush of low potassium dextran solution (Perfadex^®^). The transplantation was carried out through a clamshell incision. Explantation of the native lungs was protracted and difficult due to dense intra-pleural adhesions. Implantation was performed with the standard technique. The right lung was implanted first, using continuous 3/0 polypropylene sutures for the bronchial anastomosis (end-end-technique), continuous 4/0 polypropylene sutures for the pulmonary vein to left atrial anastomosis, and continuous 5/0 polypropylene sutures for the pulmonary arterial anastomosis. According to the standard technique, the donor bronchus was left as short as possible, and the sutures were applied almost level with the upper lobe division. The left lung was implanted in a similar fashion. The overall ischemia time was 6 hours and 8 minutes for the right lung, and just over 8 hours for the left. The patient had a persistent left-sided superior vena cava, which was carefully dissected away from the pulmonary vessels. Despite measures to reduce bleeding, including the use of intra-operative veno-arterial extra-corporeal membrane oxygenation (ECMO) instead of cardiopulmonary bypass, generous use of blood product replacement therapy and full dose of aprotinin, he bled excessively from the pleural bed. Because of persistent bleeding, the chest was packed at the end of operation with skin closure. He was given routine immunosuppressive therapy including induction with rabbit anti-thymocyte globulin (RATG) and intravenous methyl prednisolone (three doses of 125 mg each at 8 hourly intervals, followed by a tapering regimen from 1 mg/kg/day) according to our standard unit protocol.

During the immediate post-operative period, he was re-explored twice due to bleeding. After correction of the abnormal coagulation with fresh frozen plasma, cryoprecipitate and platelet transfusions, he was also given 3.6 mg of recombinant activated factor VII. Eventually, the bleeding settled and the chest was closed on the 4^th ^post-operative day. Subsequent surveillance bronchoscopies showed slough at the bronchial anastomoses with thick purulent secretions in the distal airways bilaterally. A percutaneous tracheostomy was performed on the 9^th ^post-operative day due to prolonged requirement for ventilatory support and to aid in bronchial toilet, and the patient weaned successfully from mechanical ventilation. The bronchoalveolar aspirate revealed *Pseudomonas aeruginosa *and *Candida parapsilosis*, which were treated appropriately.

**Figure 1:**
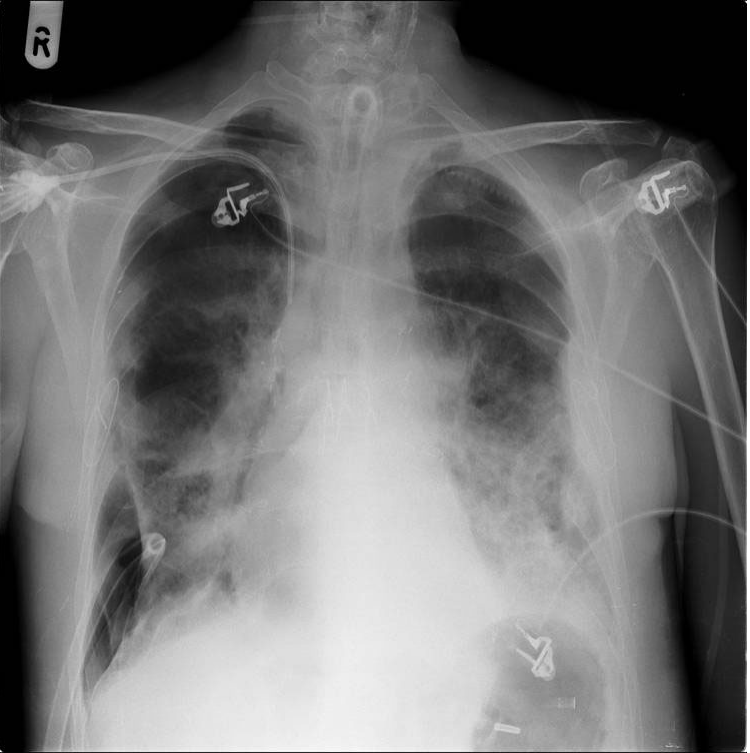
Chest X-ray showing right pneumothorax with an intercostal drain in situ.

**Figure 2:**
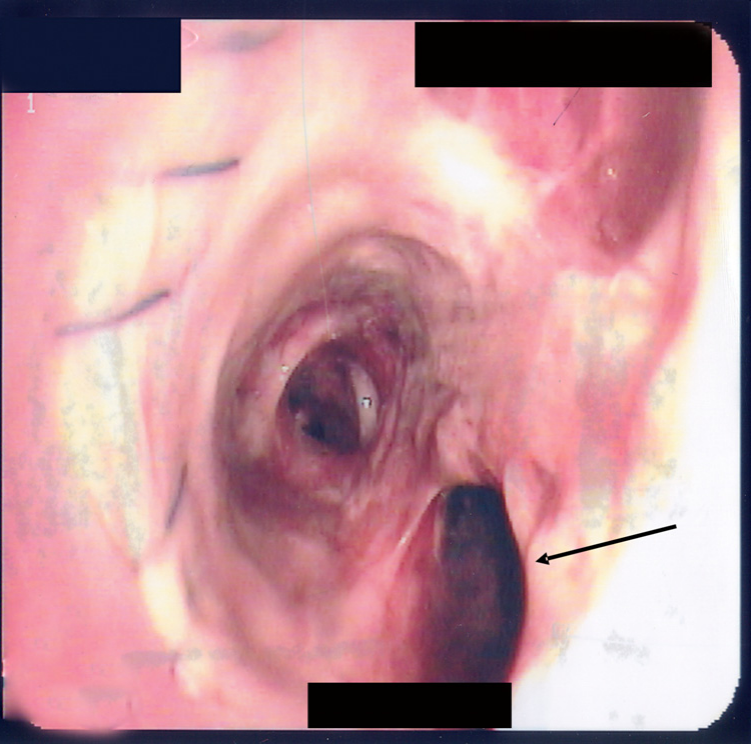
Bronchoscopy of right main bronchus with arrow pointing to the hole in the donor bronchus just distal to the right bronchial anastomosis.

On the 14^th ^post-operative day, while breathing spontaneously, the patient suddenly developed a right pneumothorax, which was treated with a right intercostal drain [Fig 1]. Bronchoscopy revealed a sizable hole in the donor right main bronchus distal to anastomosis [Fig 2]. Having been previously stable, his condition rapidly deteriorated over the next 24 hours, with pyrexia, requirement of ventilatory support and signs of systemic sepsis. CT scan could not be performed due to the clinical instability. A prompt decision was made to repair the bronchopleural fistula.

**Figure 3:**
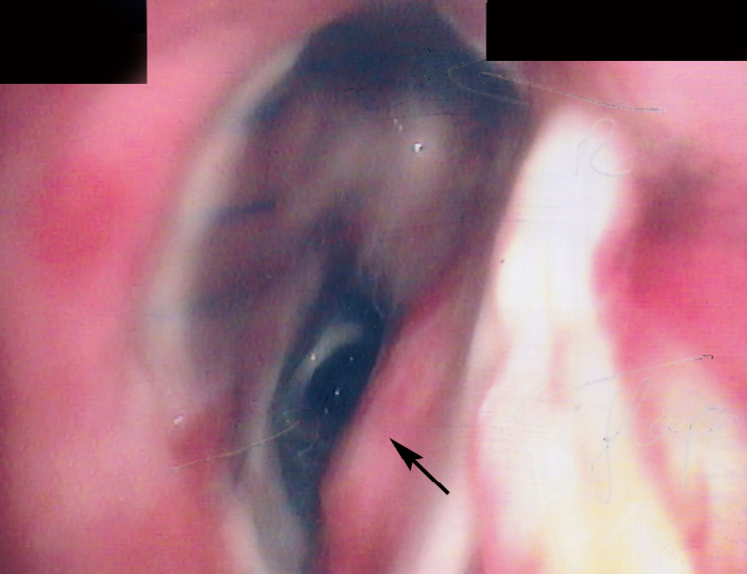
Bronchoscopy following repair of the fistula. The arrow points to the pericardial flap.

**Figure 4:**
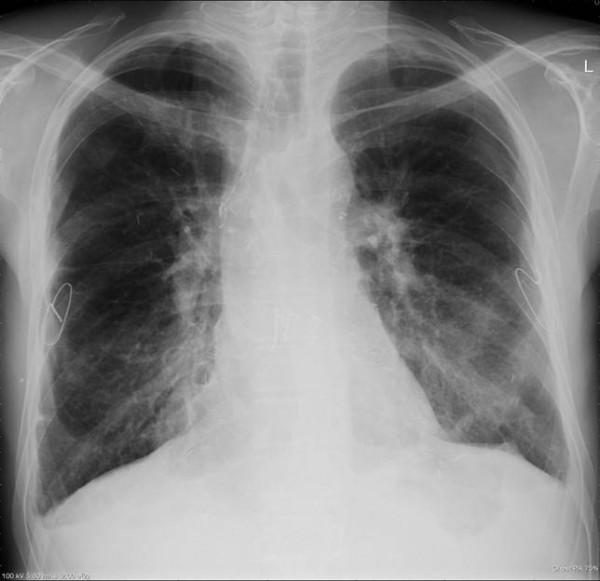
Chest X-ray 4 months following repair of the bronchopleural fistula.

Single lung ventilation was attempted using left-sided double lumen endo-tracheal tube but not tolerated due to poor oxygenation, with oxygen saturations reducing to 85% on an FiO2 of 1. The patient was not in a suitable state to undergo the surgical procedure; therefore veno-venous ECMO was established using Levitronix Centrimag^® ^[Waltham, Massachusetts]. The right internal jugular vein was cannulated with a 14 Fr Bio-Medicus^® ^DLP long cannula [Medtronic, Minneapoplis] for the oxygenated return, and the right common femoral vein was cannulated with a 17 Fr Bio-Medicus^® ^DLP long cannula [Medtronic, Minneapoplis] for the deoxygenated venous blood. Venous cannulation was performed percutaneously under the guidance of trans-oesophageal echocardiography to ensure adequate positioning of the cannulae. A single intravenous dose of 5000 units of heparin was given before the start of ECMO. We utilized a HILITE^® ^7000 LT oxygenator [Medos Medizintechnik AG] with a Levitronix Centrimag^® ^[Waltham, Massachusetts] pump. There was an immediate improvement in gas exchange. The oxygen saturations improved to 97%, with a pO2 10.6 kPa and pCO2 of 4.25 kPa. The ECMO flow rate was maintained at 2.8 L/min, with the rotor at 3500 RPM (revs per min). We proceeded with a right posterolateral thoracotomy. A 2 cm fistula was seen in the donor right main bronchus, involving the membranous part posteriorly. The fistula was closed with a large pericardial flap using interrupted 3/0 polypropylene suture, reinforced with an intercostal pedicle flap, which was dissected at the time of thoracotomy. The pleural space was cleaned thoroughly and all debris was washed prior to chest closure. The double lumen endotracheal tube was removed following the operation and ventilation was maintained using the single lumen tracheostomy tube, on a pressure controlled ventilation mode with a positive end-expiratory pressure of 2 mm Hg and a peak pressure of up to 18 mm Hg at 12 breaths per minute. At the same time 100 % oxygen was delivered in the ECMO circuit. Post-operative bronchoscopy revealed excellent closure [Fig 3] and pleural space air leak ceased completely post-operatively [Fig 4].

## Results

The patient did very well. ECMO was maintained for 6 days after repair of the bronchopleural fistula and regular surveillance bronchoscopy demonstrated intact anastomoses and satisfactory right main bronchus calibre. No further heparin was given, and the activated clotting time (ACT) was kept between 140 and 160 seconds. There were no bleeding problems. Pressure controlled ventilation was maintained using the single lumen tracheostomy tube. The peak airway pressures were gradually increased to 20–25 mm Hg at the time of removal of ECMO. The oxygen delivered via ECMO was adjusted according to the arterial blood gas results, and was successfully reduced to 40% within 4 days. After the first 48 hours, the ECMO flow rate was maintained at 2.5 L/min, with 3200 RPM. Prior to discontinuation of ECMO, the patient was relying on his lung for oxygenation with no oxygen given through the oxygenator. Both the cannulae were successfully removed with application of pressure on the site and without any problems. He was discharged home 59 days after the original transplant, with an FEV1 of 2.01 litres and a normal flow volume loop and he remains well 9 months later.

## Discussion

Bilateral sequential lung transplantation is now an accepted therapeutic option for selected patients with Cystic Fibrosis-related end-stage respiratory failure [2]. However, airway complications can result in significant problems in these patients. Earlier transplants in 1980's had a high incidence of life threatening anastomotic dehiscence. With refined surgical techniques the frequency of such events has diminished considerably [3-5]. While the overall incidence of significant airway complications is reported at 10–15% following lung transplantation, the risk of disastrous complications like anastomotic dehiscence and bronchopleural fistula remains low [Table 1]. The incidence of BPF in our unit is under 1% in over 250 cases of lung transplantation, involving more than 300 bronchial anastomoses (unpublished data).

**Table 1:** Previous reports of bronchial dehiscence following lung transplantation.

Reference	Total no. of lung transplants	Total no. of anastomoses	Total no. of airway complications	No. of bronchial dehiscence
Kshettry V R [1]	102 (77 SLT, 25 BSLT)	127	19 (15%)*	3 (2.3%)*
Alvarez A [3]	100 (29 SLT, 61 BSLT)	151	8 (5.3%)*	1(0.6%)*
Ruttmann E [4]	81 (29 SLT, 48 BSLT)	125	16 (12.8%)*	1(0.8%)*
Van de Wauwer C [5]	232 (102 SLT, 130 BSLT)	362	57 (15.7%)*	29 (8.0 %)*

SLT: Single lung transplant, BSLT: Bilateral sequential lung transplant.

* denotes the percentage of complications in total anastomoses.

Several factors have been implicated in the aetiology of bronchopleural fistula following lung transplantation. Bronchial ischaemia is considered the major contributor, as the bronchial circulation is lost during harvest and the viability of the donor bronchus is initially dependent upon retrograde low-pressure collaterals derived from the pulmonary artery [6]. Our case had a bleeding problem post-operatively, and required re-exploration and packing. The pressure of the packs or clots might have exacerbated ischaemia of the donor bronchus i.e. pressure necrosis of the right main bronchus distal to the anastomosis. In addition, we used activated factor VII to correct coagulopathy. The use of activated factor seven had been associated with the development of acute myocardial infarction [7] but an association with the development of bronchial ischaemia and dehiscence following lung transplantation has not been reported.

To prevent ischemia of the donor bronchus, several anastomotic techniques have been described, such as shortening the donor bronchus [8], end-end or telescopic anastomosis, direct revascularization of the donor bronchial arteries and wrapping a vascularised omental or internal mammary artery flap around the bronchial anastomosis [9]. As the donor bronchial segment is dependent on retrograde pulmonary blood flow, it seemed logical that donor ischemia would be minimized by keeping the donor bronchus as short as possible. This has been supported by clinical experience with double lung transplantation with bilateral bronchial rather than tracheal anastomoses [10]. We also keep the shortest possible length of the donor bronchus. Initially the telescoping technique was thought to prevent bronchial anastomotic complications [6]; however recent evidence suggests that the end-end anastomosis technique is associated with significantly less airway complications [5]. Similarly the use of running sutures in the membranous part and interrupted stitches for the cartilaginous portion has conventionally been advocated; however, recent evidence has shown that the single running suture technique with monofilament sutures used for bronchial anastomosis in lung transplantation provides optimal results with regards to early and long-term bronchial healing [11]. We have used continuous running sutures as a standard for bronchial anastomosis at our institution for over 10 years. This is the first case of bronchial disruption in our experience, involving the membranous portion of the donor bronchus, distal to the anastomosis.

Direct bronchial artery revascularization has been described by some authors [12,13], achieving complete revascularization in up to 60% of patients. However, due to the added complexity and equal incidence of complication rates, there is currently little evidence to support its use. Wrapping the bronchial anastomosis with omentum or other vascularized pedicles has demonstrated reestablishment of collateral circulation to the donor bronchus [14]. However, omentopexy adds substantial complexity and morbidity to lung transplantation due to the addition of a laparotomy and an obligatory diaphragmatic defect. This technique is not infallible and has not been advocated by some investigators [15]. Other vascularized flaps have been employed to obviate omentopexy [16]; however, some investigators have shown satisfactory bronchial healing without any vascularized coverage of the bronchus [17]. We do not utilise the technique of anastomotic wrapping in our centre.

The perioperative use of steroid therapy was believed to be deleterious for the bronchial anastomosis [18], however recent evidence suggests that low to moderate doses of pre- and post-operative steroids do not affect bronchial healing [3]. Our patient was not on maintenance prednisolone prior to transplant.

Infections, particularly due to aspergillus, have been associated with impaired bronchial healing [6] and a higher rate of airway complications have been reported in patients with septic lung disease (cystic fibrosis) [1]. Our case showed evidence of bronchial infection with *Pseudomonas aeruginosa *and *Candida parapsilosis*, which might have contributed to the development of BPF.

Management of BPF in lung transplant recipients is very challenging with limited published experience in the English literature. With few exceptions, most reports pertain to management of post pneumonectomy BPF in lung cancer patients. The approach will depend on a number of factors such as the size and site, time between surgery and presentation, whether or not empyema is present and the patient's general health status. The key points of BPF management include empyema drainage and infection control, fistula closure and reinforcement, and pleural space obliteration. Surgical closure techniques include direct stump closure with flap reinforcement, trans-sternal bronchial closure, thoracoplasty with or without extra-thoracic chest wall muscle transposition and video-assisted thoracoscopy. Kirk *et al *described the surgical management of bronchial dehiscence which occurred despite using an omental flap in a case of lung transplantation for pulmonary fibrosis [19]. They excised the necrotic bronchus and re-anastomosed the distal healthy bronchus using interrupted polypropylene sutures. They constructed the second anastomosis at the level of bifurcation of the donor left main bronchus, to achieve perfusion from pulmonary bronchial collaterals. They also wrapped a vascular pericardial flap around the anastomosis. We utilised pedicled pericardial flap to close the BPF, and the intercostal flap to reinforce the repair.

In high-risk surgical patients, endoscopic repair may serve as the only therapeutic option. In 1977 Ratliff *et al *reported the first successful endo-bronchial management of BPF using tissue glue and a lead shot [20]. Since then, many reports using different devices have appeared. These include ethanol silver nitrate, cyanoacrylate compounds, coils, lead plugs, balloons, fibrin or tissue glue, antibiotics, gel foam, spigots, and autologous blood patch. Recently Mora *et al *described a series of 18 patients, including 1 post-lung transplant, treated endoscopically for BPF [21]. The fistula was 9 mm in size, and appeared 30 days post-transplant. It was successfully closed by 2 injections of fibrin sealant (Tissucol^®^) through the catheter of the fiberoptic bronchoscope without any complications. In our case, the size of the fistula prohibited bronchoscopic approach to treatment.

Borro *et al *described a case of spontaneous closure of bronchial fistula to the mediastinum in a heart-lung transplant patient [22]. They suggested that since the bronchial perforation in their patient occurred in the 3^rd ^post-operative week, full cicatrisation of the mediastinum helped limit the spread of infection and contributed to the favourable outcome obtained by adequate antibiotic and physiotherapeutic treatment.

Achieving adequate ventilation is often difficult in these patients, particularly if single lung ventilation has to be achieved. The use of differential ventilation using double-lumen endotracheal tube and jet ventilation or cardiopulmonary bypass has been the traditional options [19]. We utilised veno-venous ECMO, which is now an established tool in our centre for managing patients with primary graft failure after lung transplant. With improved technology it is now possible to use this device with minimal heparinization, thereby preventing any bleeding complications [23,24]. Our patient had severe intraplerual adhesions, causing bleeding following explantation of the native lungs. Therefore, based on the available evidence at that time [23], we decided to use intra-operative veno-arterial ECMO to avoid full heparinization otherwise necessary with conventional cardiopulmonary bypass. However, we encountered bleeding problems post-operatively, requiring blood products as well as activated factor VII. Recently published data also suggests that intra-operative use of ECMO may be associated with increased risk of bleeding [25].

During the first 24 hours following the repair of BPF, the patient required full ECMO support with 100% oxygen delivery as the aim was to rest the lungs and reduce the pressure on the repair. Afterwards, the ECMO requirement diminished. We, however, used ECMO for 6 days as we believed that healing of the repaired bronchus would be sufficient around that time. During this period we carried out frequent flexible bronchoscopic assessments. At day 5, the anastomosis appeared to be healing well.

To our knowledge this is the first reported case for using ECMO as a bridge for successful surgical repair of BPF following lung transplantation utilising pericardial and intercostal flaps. We have used the Levitronix Centrimag device, which was maintained without any anticoagulation for six days after repair of the fistula (ACT was kept between 140–160 seconds). There were no clots in the system on full examination when it was finally removed.

## Conclusion

In summary, the combination of an intercostal and a pedicled pericardial flap provides adequate robust vascularised tissue for sealing a large BP fistula following lung transplantation. Veno-venous ECMO allows a feasible bridge to recovery.

## Competing interests

The author(s) declare that they have no competing interests.

## Authors' contributions

NY performed the operation. NUK assisted in the operation and took care of the patient in the ward along with NK and AM. NK and AM were also involved in re-exploration. MA and CL performed bronchoscopies and were involved with immunosuppression, ward care and the follow up.

All authors read and approved the final manuscript.
